# The primate-specific peptide Y-P30 regulates morphological maturation of neocortical dendritic spines

**DOI:** 10.1371/journal.pone.0211151

**Published:** 2019-02-13

**Authors:** Janine R. Neumann, Suvarna Dash-Wagh, Alexander Jack, Andrea Räk, Kay Jüngling, Mohammad I. K. Hamad, Hans-Christian Pape, Michael R. Kreutz, Martin Puskarjov, Petra Wahle

**Affiliations:** 1 Ruhr-Universität Bochum, AG Entwicklungsneurobiologie, Fakultät für Biologie und Biotechnologie, Bochum, Germany; 2 Institut für Physiologie I, Westfälische Wilhelms-Universität Münster, Münster, Germany; 3 Neuroplastizität, Leibniz-Institut für Neurobiologie, Madgeburg, Germany; 4 Leibniz Group 'Dendritic Organelles and Synaptic Function', Center for Molecular Neurobiology, University Medical Center Hamburg-Eppendorf, Hamburg, Germany; 5 Molecular and Integrative Biosciences, Faculty of Biological and Environmental Sciences, University of Helsinki, Helsinki, Finland; Universita degli Studi di Torino, ITALY

## Abstract

The 30-amino acid peptide Y-P30 corresponds to the N-terminus of the primate-specific, sweat gland-derived dermcidin prepropeptide. Previous work has revealed that Y-P30 enhances the interaction of pleiotrophin and syndecans-2/3, and thus represents a natural ligand to study this signaling pathway. In immature neurons, Y-P30 activates the c-Src and p42/44 ERK kinase pathway, increases the amount of F-actin in axonal growth cones, and promotes neuronal survival, cell migration and axonal elongation. The action of Y-P30 on axonal growth requires syndecan-3 and heparan sulfate side chains. Whether Y-P30 has the potential to influence dendrites and dendritic protrusions has not been explored. The latter is suggested by the observations that syndecan-2 expression increases during postnatal development, that syndecan-2 becomes enriched in dendritic spines, and that overexpression of syndecan-2 in immature neurons results in a premature morphological maturation of dendritic spines. Here, analysing rat cortical pyramidal and non-pyramidal neurons in organotypic cultures, we show that Y-P30 does not alter the development of the dendritic arborization patterns. However, Y-P30 treatment decreases the density of apical, but not basal dendritic protrusions at the expense of the filopodia. Analysis of spine morphology revealed an unchanged mushroom/stubby-to-thin spine ratio and a shortening of the longest decile of dendritic protrusions. Whole-cell recordings from cortical principal neurons in dissociated cultures grown in the presence of Y-P30 demonstrated a decrease in the frequency of glutamatergic mEPSCs. Despite these differences in protrusion morphology and synaptic transmission, the latter likely attributable to presynaptic effects, calcium event rate and amplitude recorded in pyramidal neurons in organotypic cultures were not altered by Y-P30 treatment. Together, our data suggest that Y-P30 has the capacity to decelerate spinogenesis and to promote morphological, but not synaptic, maturation of dendritic protrusions.

## Introduction

A distinctive feature of the primate cerebral cortex among mammals is a large number of supranumerary synapses. In the human prefrontal cortex, for instance, overproduction and elimination of dendritic spines and synaptic sites continues into the third decade of life [1, 2]. Overproduction of spines or their impaired elimination has been linked to neurodevelopmental disorders [3]. However, the molecular signaling mechanisms regulating the maturation of dendritic protrusions are not fully characterized.

The 30 amino acid peptide Y-P30 corresponds to the N-terminus of the primate-specific dermcidin prepropeptide (encoded in humans by the *DCD* gene), which is produced mainly by eccrine sweat glands of the skin [4, 5]. The peptide, also termed ‘survival promoting peptide’, has been originally identified as promoting the survival of cortical neurons after stab wounding [6, 7], to protect hippocampal neurons against oxygen and glucose deprivation [8], and to partially rescue retinal ganglion cells from death after optic nerve crush [9]. Furthermore, Y-P30 has been reported to enhance the migration of human T24 bladder carcinoma cells, PC12 cells and primary astrocytes from rat cortex [10]. Y-P30 is a ligand which enhances the interaction of the neurite growth-promoting matrix protein pleiotrophin and membrane-bound syndecan-2 and syndecan-3 [11]. Syndecan-3 is enriched on axons, and Y-P30 has been shown to promote axonal growth in a syndecan-3-dependent and heparan-sufate side chain-dependent manner in cortical stem cell-derived neurons, and in days-in-vitro (DIV) 3–4 retinal and cortical microexplants [11–13]. In immature neurons, ligand binding to syndecans, including that of Y-P30, activates Y418 c-Src phosphorylation and evokes a Src-dependent activation of p42/44 ERK kinase [13, 14]. Y-P30 increases the levels of active Src kinase and F-actin in cortical axonal growth cones, leading to their stabilization against collapse-inducing semaphorin-3a [13]. With ongoing development, syndecan-2 becomes the dominant isoform being enriched in dendritic spines [15, 16]. Overexpression of syndecan-2 for seven days in immature embryonically dissociated hippocampal neurons results in an acceleration of morphological maturation of spines starting from preexisting filopodia: The fraction of protrusions with heads, aka mature-like spines, increases from 20–25% in control to 75%, a value normally seen in DIV 30 hipoocampal cultures; moreover, the protrusions become shorter indicating a higher degree of maturity [17]. The density of protrusions remains at 20–25 per 100 μm dendrite [17]. Further, in syndecan-2 overexpressing hippocampal neurons the spine maturation proceeds without enhancement of synaptogenesis, as indicated by the lack of association of syndecan-2-positive spines with synapsin-1 positive presynaptic boutons [17]. Moreover, the developmental steps are influenced by the plating density of the cells [17] and in addition, by calcium signaling [18]. Mechanistically, syndecan-2 becomes phosphorylated by EphB2 and signals via the calcium/calmodulin-dependent serine kinase (CASK) to trigger the transformation of filopodia into mature spines [17–21].

Here, we analyzed dendritic differentiation of postnatal rat cortical neurons in organotypic culture (OTC), a 3D spontaneously active culture system characterized by an in vivo-like degree of cellular complexity and wiring. In DIV 10 and DIV 20 pyramidal neurons Y-P30 had no effect on dendritic arborization patterns. However, the density of protrusions along apical, but not basal dendritic branches was lower with shorter protrusions and less filopodia. This suggested that Y-P30 slows down the building of apical dendritic protrusions and promotes their morphological maturation. Opposite to the effects in immature neurons [13], Y-P30 in differentiated neurons caused a decrease in Y418 c-Src phosphorylation and no longer activated p42/p44 ERK kinase. Unexpectedly, acute Y-P30 exposure of OTC was associated with a transient reduction of NT3 mRNA expression at DIV 10, and at DIV 20 with a dephosphorylation of Y1472 GluN2B. Both, NT3 and GluN2B are crucially involved in regulating excitatory synapse development and plasticity [22–25]. A decrease in the frequency of glutamatergic miniature excitatory postsynaptic currents (mEPSCs) without a change in amplitude of kinetics was observed in DIV 15–17 principal cortical neurons grown in primary culture in the presence of Y-P30. However, amplitude and rate of calcium events recorded in DIV 14–23 pyramidal neurons in OTC were not altered by Y-P30 treatment, suggesting that the difference in protrusion density had no measurable impact on network activity.

In summary, we show that pleiotrophin-syndecan signaling undergoes developmental changes from activating to inhibiting signaling cascades, that the effect of activating syndecan signaling in more differentiated cortical neurons differs from the effects of syndecan-2 overexpression in immature hippocampal cells, and that a difference in apical dendritic spine density is not reflected by a difference in network activity.

## Materials and methods

### Y-P30

A synthetic peptide homologous to the human peptide sequence (YDPEAASAPGSGNPCHEASAAQKENAGEDP) [4] was used for the analyses. The peptide was dissolved in 5 mM Tris-HCl pH 7.4 at 100 μM concentration and stored at -20°C. Y-P30 is very stable on skin and in solution [6, 16, 26, 27]. It was applied at 1 μM final concentration in the medium. The efficiency of Y-P30 for cerebellar granule cell neurite growth was highest in the the low micromolar range [12].

### Neuronal cultures and Biolistic Transfection

Organotypic (roller-tube) cortical cultures (OTCs) were prepared from newborn P0/P1 pigmented Long Evans rats from the in-house breeding facility with approval from the Ruhr-University Animal Research Board and the State of Northrhine-Westfalia as described [28, 29, 30]. Briefly, visual cortex was explanted at postnatal day (P) 0 (day of birth) or P1, and cut into 350 μm thick slices using a McIlwain tissue chopper (Ted Pella, Redding, CA, USA). Cultures were fed three times a week with medium containing 25% adult horse serum, 25% Hank's balanced Salt Solution, 50% Eagle's Basal Medium, 1 mM L-glutamine (all from Life Technologies, Karlsruhe, Germany) and 0.65% D-Glucose (Merck, Darmstadt, Germany). To inhibit glial growth, 10 μl of an antimitotic cocktail (uridine, cytosine-β-D-arabino-furanosid and 5-fluorodeoxyuridine; each stock 1 mM; all from Sigma-Aldrich, Taufkirchen, Germany) was applied at day in vitro (DIV) 2 for 24 h. OTCs were transfected at DIV 5 with endotoxin-free (Qiagen) pEGFP-N1 plasmid encoding enhanced green fluorescent protein under the cytomegalovirus promoter (Clontech via Takara Bio Europe, Saint-Germain-en-Laye, France). DNA coating of gold particles, preparation of cartridges, and transfection were performed as described [26]. Cultures from every animal (5–6 pups prepared per batch) were allocated to the control group receiving vehicle stimulation as well as to the treatment group receiving Y-P30 (1 μM, applied with fresh medium at DIV 2 and 4 for analysis at DIV 5, at DIV 7 and 9 for analysis at DIV 10, or daily from DIV 10 or DIV 15 for analysis at DIV 15 or DIV 20, respectively). Control cultures were always handled at the same time and vehicle-stimulated with the vehicle, 5 mM Tris-HCl pH 7.4. At DIV 5, 10 and 20, the cultures were fixed and processed for EGFP or GABA immunohistochemistry as described [29]. Single pulse stimulation has been done to analyse acute effects on kinase activation and phosphorylation with 1 μM Y-P30 supplemented to the medium at DIV 5, DIV 10, and DIV 20 for 15 min and for 60 min. Further, to look for effects on neurotrophin expression, single pulse stimulation has been done over the course of 1, 2, 4, 8, 24 h at DIV 10. In both experiments Y-P30 remained in the medium for the times given. In addition, a DIV 5 to DIV 10 treatment with Y-P30 has been done to look for longterm changes in neurotrophin expression. For Western blot and RNA expression analyses, OTCs were harvested, frozen on dry ice, and stored at -80°C. OTCs used in this study were evaluated carefully to exhibit a maximum in culture integrity with no damage that would indicate an impaired culture quality.

Primary neuronal cultures were prepared from embryonic day 18 rat cortices as described [31]. Neurons were plated onto poly-L-lysine-coated glass coverslips placed into 4-well plates and were grown in Neurobasal medium containing B27 supplement, glutamine, and penicillin+streptomycin antibiotic mix (Neurobasal medium and all additives from Invitrogen/Gibco, Carlsbad, CA) at a density of 1.0×10^5^ cells/cm^2^). Treatment with 1 μM Y-P30 or equivalent volume of distilled water was started from DIV 7 and refreshed, by replacing half of the culture medium every second day, retaining a constant concentration of treatment.

### Reconstruction of neurons and spines

Pyramidal neurons from supragranular and infragranular layers and interneurons were Neurolucida-reconstructed at 1000x magnification by an experienced experimenter blinded to condition and crosschecked for correctness and classification by a second experienced experimenter, who was also blinded to condition. All pyramidal cells had axons with a clear origin at the soma or a proximal dendrite which descended into the white matter. Supragranular pyramidal cells had apical dendrites extending into layer I. Infragranular pyramidal neurons were included when apical dendrites ended in middle layers. Length of apical dendrites, average length of basal dendrites and the number of segments were determined. Multipolar interneurons with smooth or sparsely spinous dendrites and locally branching axons were sampled from layers II-VI. Total and mean dendritic length and segments, and the number of primary dendrites were determined. For every condition, at least 10 OTC from different culture batches were analyzed. The number of cells analyzed is given in Fig 2 and Fig 3. Spine density and size was determined by reconstructing secondary oblique dendrites branching off the apical dendritic shaft at 1400x magnification with a Camera Lucida by trained observers blinded to condition as previously described [29]. Apical shafts and tufts were excluded. Spine density along basal dendrites was sampled in the mid-portion avoiding the proximal 30 μm and the distalmost tips; 3 dendrites and a total of 300 μm dendritic length were assessed per cell. The number of spines per 100 μm dendrite length was calculated. Spines were defined as protrusions with distinct heads, and sampled as mushroom/stubby spines with large heads and as thin spines with small heads following established criteria [32, 33]. Filopodia were defined as fine protrusions without a distinct head. GABA cell soma are was determined from OTC chronically exposed to Y-P30, or vehicle, or NT4 (20 ng/mL) as positive control; the outline GABA-immunoreactive somata was drawn at 1000x magnification with a Camera lucida; 50–80 somata per OTC were sampled in a perpendicular strip from the pial surface down to the white matter. Graphs showing means with SEM were constructed with SigmaPlot 12.1. The number of neurons assessed is reported in the figures.

### Protein blots

OTC were picked from the coverslips, frozen individually, lysed with 20 μl RIPA-SDS buffer, and boiled. Lysates of one OTC were loaded per lane on 12x12 cm 10% acrylamide gels. Nitrocellulose membranes strips of the appropriate size were placed horizontally to cover the molecular weight range of the target protein of interest, and blotted as described [29]. Up to 8 membrane strips from each gel could be incubated with appropriate antibodies. This way, the proteins not altered in Y-P30 exposed OTC serve as additional controls, besides the house-keeping proteins β-actin or β-tubulin, for those epitopes which were altered by the treatment. Visualization of protein bands was done with 5-bromo-4-chloro-3-indolyl-phosphate/nitro blue tetrazolium (BCIP/NBT; Promega, Mannheim, Germany). Phospho-MAPK detection was achieved using chemoluminescence (CPD-Star, New England Biolabs, Frankfurt, Germany), followed by stripping and reprobing for MAPK total protein detection. To assess phosphorylated synaptic proteins, 50% of a lysate of one OTC was loaded on the right and the left half of a gel (always pairing control and treatment); one half was developed for the total protein, the other half for the phosphorylated epitope of the protein; both were normalized to β-actin from each lane. Membrane strips were scanned and quantified (UV solo BioDoc; Biometra). For Figs 3H and 4D, representative bands of proteins developed in a given gel had been cropped from the scanned images of the membrane strips and presented together with the house-keeping protein band from these lanes. S1 Table lists all antibodies and reagents including the sources used in the study.

### Real-time qPCR and reverse transcriptase PCR

At DIV 10, 5 OTCs (vehicle-stimulated or treated with 1 μM Y-P30) were picked from the coverslips, frozen on dry ice, pooled, fresh and stored at -80°C until RNA extraction. Total RNA was isolated by using the RNeasy Mini Kit (Qiagen). RNA purity and quantity was measured by spectrophotometry (BioPhotometer plus, Eppendorf) and visualization of ribosomal RNAs (28S, 18S) by gel electrophoresis. Residual genomic DNA was digested by DNase I (0.34 U/μL, Qiagen) treatment. cDNA libraries were generated from 1 μg RNA using the First strand cDNA synthesis kit (Fermentas) with M-MulV-Reverse Transcriptase (2 U/μL) and random hexamer primers (5 μM) at 37°C for 60 min. Real-time qPCR was performed using the realplex2 system (Eppendorf) with white 96-well plates sealed with cap strips (Eppendorf) by measuring the incorporation of the fluorescent dye SYBR green. For each reaction (20 μL) a master mix of 1x SYBR-Mastermix (Mesa Blue qPCR MasterMix Plus, Eurogentec) and 300 nM forward and reverse primers (resp. 150 nM for 18S primers, Eurogentec) was prepared. The PCRs were optimized for the following cycle conditions: 95°C 15 s, 60°C 20 s, 72°C 40 s and run for 40 cycles. After the PCR, a melting curve analysis was performed to confirm the specificity of the PCR, the amplified sequences have also been visualized on a 1.5% agarose gel. For each sample, triplicates were run in a PCR reaction whereas for each triplicate 2 μL cDNA was used. Specific primers for syndecan-2, syndecan-3, reelin, 18S ribosomal RNA and glucose-6-phosphate dehydrogenase (G6PDH) are given in S2 Table. The 18s RNA and G6PDH were determined in preliminary qPCR experiments to be unchanged between control and Y-P30-treated OTCs, and used as reference genes. For analysis, only PCR reactions were considered where the negative controls (e.g. “no template” controls and reverse transcription control) were negative. All reactions were performed on one single 96-well plate. The relative product quantities were determined using the E-ΔΔCt method [34] by calculating PCR-efficiencies of every amplification curve in LinRegPCR [35]. Considering PCR efficiencies, corrected Cq-values have been used to determine the relative expression of the target gene compared to the expression of the reference gene and the expression levels in control (untreated) by using REST (Qiagen).

Semi-quantitative reverse transcriptase (RT)-PCRs were performed as described [36]. The mRNA was prepared from lysates of five pooled OTCs (vehicle-stimulated or treated with 1 μM Y-P30) using a Dynabead mRNA Direct Kit (Dynal). cDNA libraries were synthesized with Sensiscript reverse transcriptase (20 U/μL; Qiagen) at 37°C for 60 min. PCR was performed in a total volume of 50 μL with Taq DNA Polymerase (0.5 U/μL; Qiagen) or Crimson Taq DNA Polymerase (0.025 U/μL; New England Biolabs). The single-band amplicons and PCR conditions are listed in S2 Table. PCR conditions were kept within the linear range determined for every product. The relative mRNA expression was determined from three PCR reactions run with cDNA libraries from at least three independently prepared cDNA libraries.

### Electrophysiological recordings

Miniature excitatory postsynaptic currents (mEPSCs) were recorded in whole-cell voltage-clamp configuration at room temperature (22 ± 1°C) from DIV 15–17 pyramidal-shaped neurons in primary culture. The extracellular recording solution contained (in mM) NaCl 124, KCl 3, CaCl_2_ 2, NaH_2_PO_4_ 1.1, MgSO_4_ 1.3, HEPES 20, D-glucose 10, bicuculline 0.01, and tetrodotoxin 0.002, pH 7.4 was adjusted with NaOH. Patch pipettes were fabricated from borosilicate glass, and their resistances ranged from 4 to 6 MΩ. The pipette solution consisted of (in mM) KCl 6, K-gluconate 123, CaCl_2_ 0.5, BAPTA 5, Mg-ATP 2, HEPES 10, D-glucose 10, NaOH 2, pH 7.3 was adjusted with KOH. A liquid junction potential (11 mV) measured with a 3 M KCl reference electrode was corrected online. Membrane potential was held at -65 mV. Access resistance was monitored throughout the recording and only cells with less than 20% change in this parameter during the recording epoch were accepted for analysis. mEPSCs recordings were analyzed using the Mini Analysis Program (Synaptosoft). Y-P30 at 1 μM or vehicle was added also to the recording solution. Recordings and analyses were made blind to experimental condition.

### Calcium imaging

For confocal calcium imaging, OTCs were gene-gun transfected with plasmids encoding CMV-promoter driven GCaMP6m and mCherry at DIV 8. Subsequently OTCs received one pulse of Y-P30 daily at 1 μM final concentration or vehicle as control. At the day of recording, the cultures received their last treatment at least 90 min prior to recording. Pyramidal neurons were first identified under the mCherry fluorescence and recorded afterwards in the green channel under constant perfusion with 32°C oxygenated ACSF (containing in mM: 125 NaCl, 5 KCl, 2 CaCl2, 1 MgSO4, 25 NaHCO3, 1.25 NaH2PO4, 25 glucose, pH 7.4), at a rate of 3–5 ml per min. Imaging was done using a Leica TCS SP5 confocal microscope (Leica, Mannheim, Germany) with a 10x objective at 1400 Hz and 371 ms/frame. In order to allow comparison of multiple cells from several batches, the normalization to the mean fluorescence intensities (Δ F/F^0^) was done as described [29, 30].

### Statistical analysis

All results are reported as mean ± SEM. Analyses were performed using SigmaPlot 12.1 (Systat Software, Inc, Erkrath, Germany). The experimental groups were compared using t-test or non-parametric Mann-Whitney U-test and one-way analysis of variance where appropriate. Statistical significance was defined as p < 0.05.

## Results

### Y-P30 does not alter somatodendritic differentiation of cortical neurons

**Fig 1:**
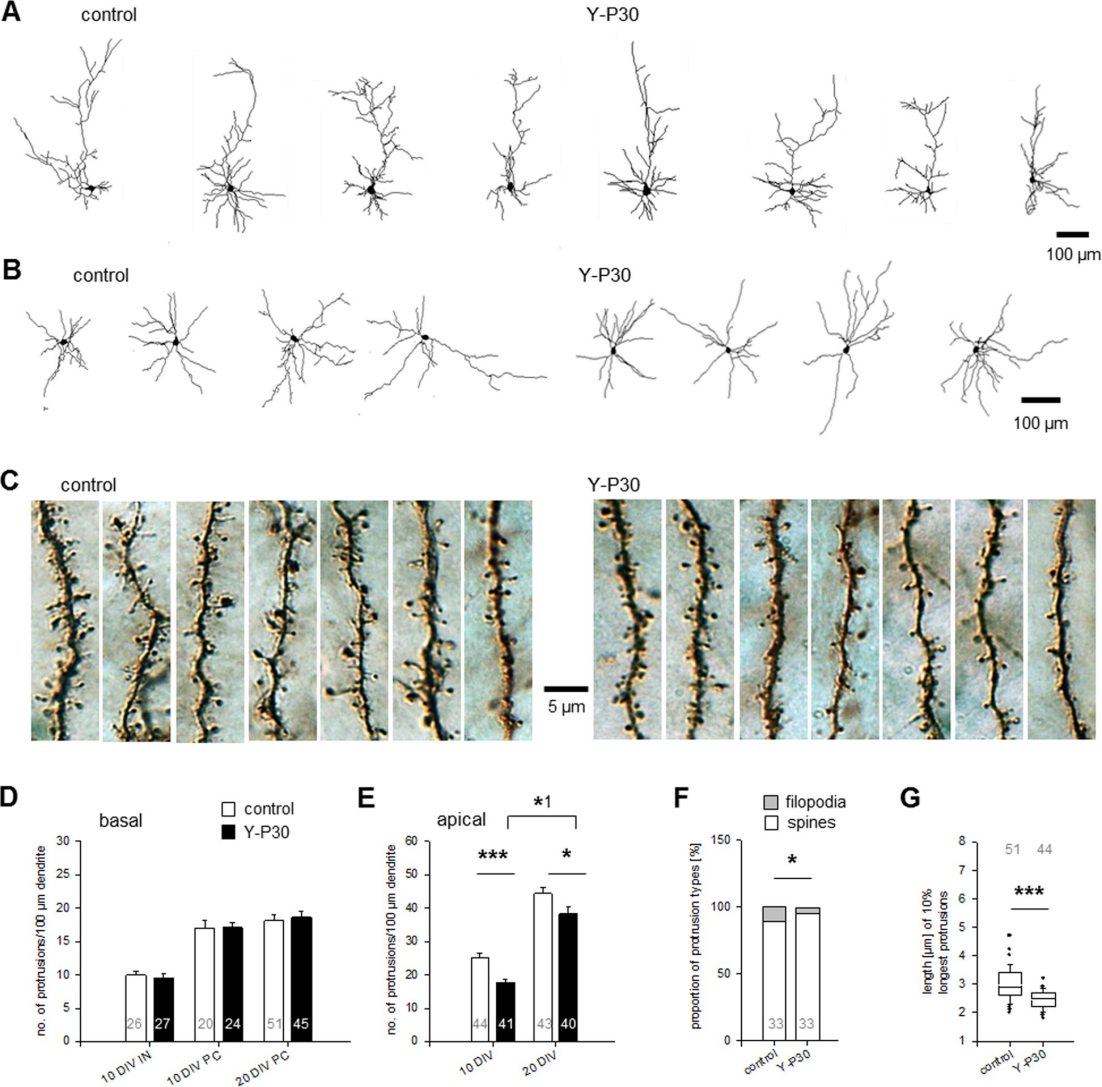
Y-P30 does not alter somatodendritic differentiation of cortical neurons but promotes filopodia pruning and morphological maturation of spines on pyramidal neurons. (A) Chronic treatment with Y-P30 had no effect on pyramidal cell dendritic growth; gallery of 4 representative cells shown per condition at DIV 10; for quantitative data see Fig 2, for graphs see S1 Fig. (B) Chronic treatment with Y-P30 had no effect on interneuronal dendritic growth; gallery of 4 representative cells shown per condition at DIV10; for quantitative data see Fig 3, for graphs see S2 Fig. (C) Segments of secondary oblique branches from the apical dendrite of seven 20 DIV pyramidal cells from control OTC and OTC treated with Y-P30. EGFP transfection at DIV 14; Y-P30 treatment with 1 μM daily from 15–19 DIV, controls were mock-stimulated daily with an equivalent volume of vehicle. Segments were ordered from left-to-right from high-to-low protrusion density to document variability; note the longer protrusions in the control group. (D) Y-P30 did not alter the density of interneuronal (IN) dendritic protrusions and basal dendritic protrusions of pyramidal cells (PC) at DIV 10 (treated with 1 μM Y-P30 at DIV 7 and DIV 9) and DIV 20 (daily one pulse of 1 μM Y-P30 from DIV 15–19). Plotted is the mean number ± SEM per 100 μm dendritic length. (E) Y-P30 decreased the density of protrusions on secondary oblique branches from the apical dendrites of pyramidal cells at DIV 10 by 27% and at DIV 20 by 13%. Plotted is the mean number ± SEM per 100 μm dendritic length. Note, that the protrusion density increased between DIV 10 and DIV 20 in vehicle-treated and in Y-P30 treated neurons. The number of cells analyzed is indicated in the bars; they derived from 9–12 OTC from three independent preparations; *** p<0.001, * p = 0.017, *^1^ p = 0.02, Mann-Whitney U-test. (F) Y-P30 increased the proportion of spines with heads (mushroom/stubby and thin spines) on secondary oblique branches from the apical dendrites of pyramidal cells at 20 DIV and decreased the proportion of filopodia (* p = 0.035, Mann-Whitney U-test). Numbers in the bars indicate the number of cells analyzed each with arbitrarily selected 3–4 segments each of about 50 μm length. (G) Assessment of protrusion length on secondary oblique branches from the apical dendrites of pyramidal cells at DIV 20. Plotted are the 10% longest protrusions sampled from 2–3 dendritic segments; every value is the mean of one cell, the numbers above the boxes are the number of cells analyzed. *** p<0.001, Mann-Whitney U-test.

**Fig 2:**
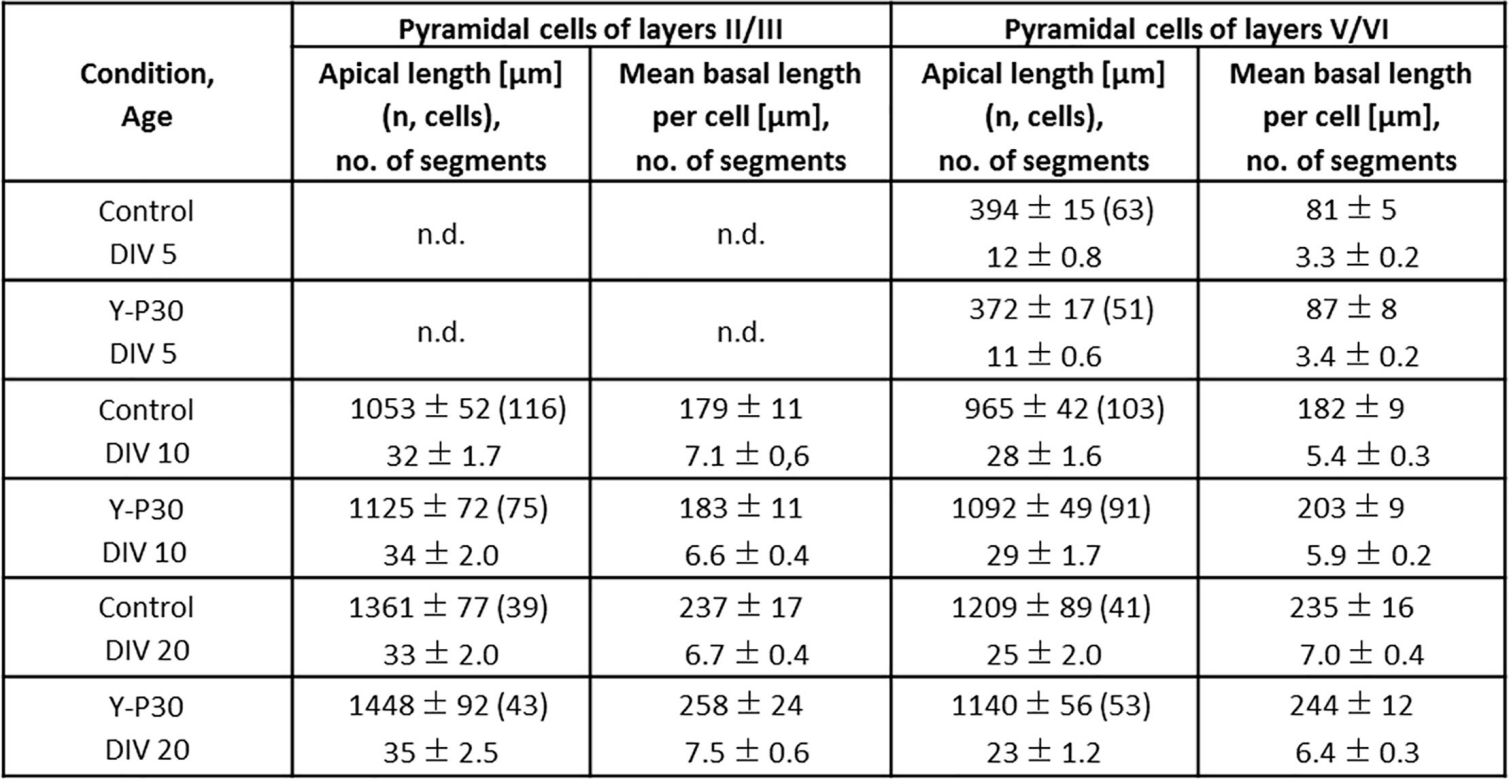
Morphometric data of layer II/III and V/VI pyramidal neurons. Cells reconstructed from DIV 5, DIV 10 and DIV 20 OTC; chronically exposed to Y-P30 or vehicle (control). Mean ± SEM, the number of cells (n) is given in brackets. At DIV 5, only cells from infragranular layers were analyzed because supragranular neurons are too immature to reliably identify the type. n.d., not determined.

**Fig 3:**
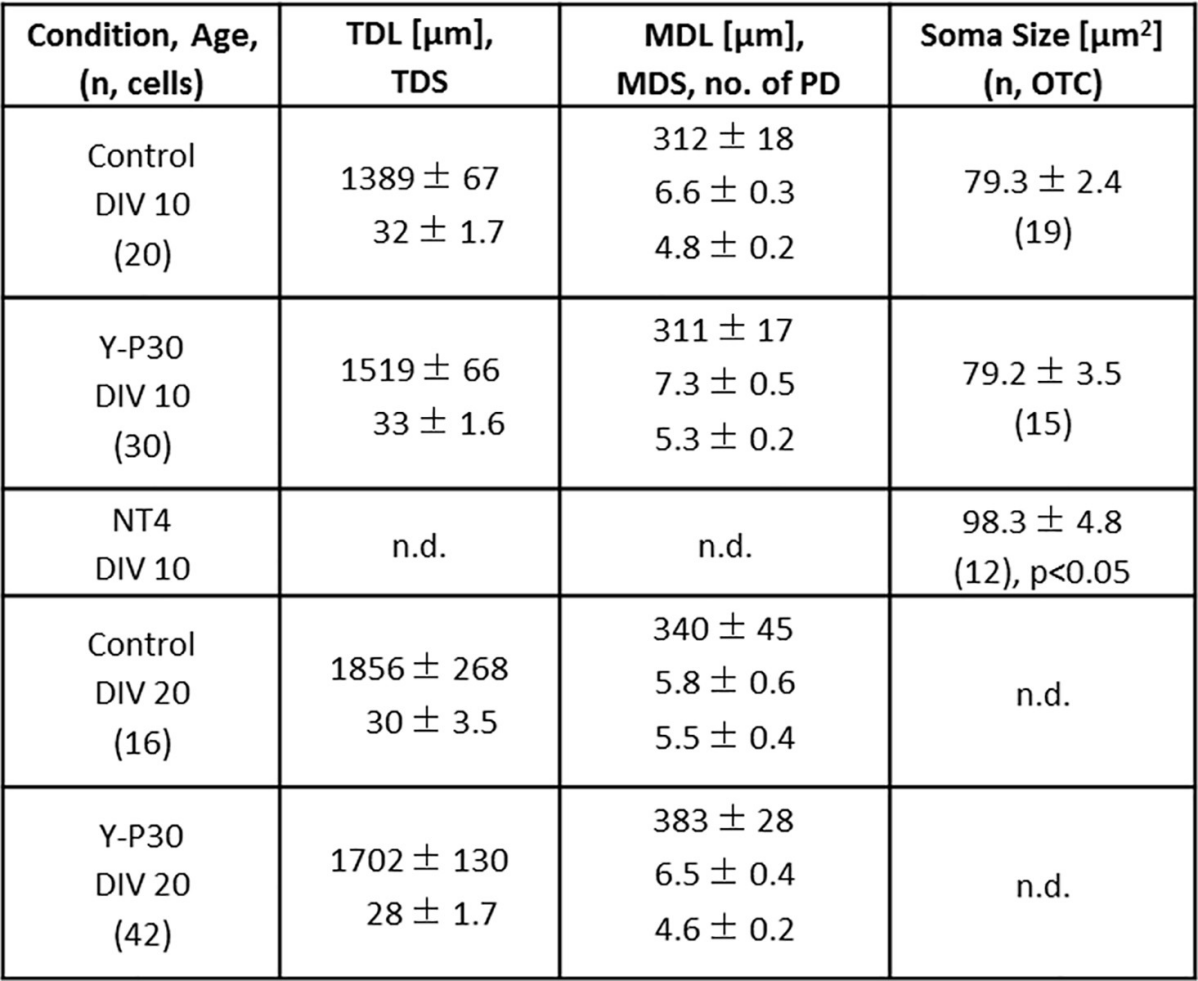
Morphometric data of multipolar interneurons. Cells reconstructed from DIV 10 and DIV 20 OTC; chronically exposed to Y-P30 or vehicle. Mean ± SEM for TDL, total dendritic length; TDS, total dendritic segments; MDL, mean dendritic length; MDS, mean dendritic segments; no.of PD, number of primary dendrites/neuron. The number of cells is given in brackets. Soma size (area in μm2 as proxy) of GABA-ir neurons is the grand average of somata analyzed in 19 and 15 OTC by sampling 50–80 somata in a perpendicular strip through all layers in every OTC. 20 ng/mL NT4 treatment has been done at DIV 10 as positive control (12 OTC) to show responsiveness of interneurons. ANOVA on ranks followed by Mann-Whitney U-test versus DIV 10 control p<0.05. n.d. not determined.

GFP-tagged Y-P30 added to the medium of dissociated cortical neurons tends to aggregate at dendritic locations [27]. However, our previous analysis of the effects of Y-P30 exposure on embryoid stem cell-derived, retinal and cortical neurons suggested that Y-P30 affects axonal but not dendritic growth [13]. To exclude that the latter could have been a false negative result due to the immaturity of neurons in short-term culture we now assessed the effects of Y-P30 exposure of postnatal rat cortical OTC grown from DIV 0–5, DIV 7–10, and DIV 15–20 with Y-P30 in the medium. Neurons were biolistically transfected with plasmids encoding EGFP, stained and reconstructed for morphometric assessment as described [28, 30]. The neurons grew substantially between DIV 5, DIV 10, and DIV 20, however, Y-P30 treatment did not alter length and branching of apical and basal dendrites of infragranular and supragranular pyramidal cells at the three ages examined (Fig 1A; Fig 2, S1 Fig). This suggested that Y-P30 has no role for elongation and branching of pyramidal cell dendrites. Y-P30 exposure did not change dendritic parameters of multipolar sparsely spinous interneurons at DIV 10 and 20 (Fig 1B, Fig 3, S2 Fig). Further, soma size of GABA-immunoreactive neurons was not different from control at DIV 10. By contrast, as a positive control, 20 ng NT4 per mL medium significantly increased the area of GABA cell soma (Fig 3, S2 Fig).

### Y-P30 promotes the morphological maturation of apical dendritic protrusions

Heparan sulfate proteoglycans are important players in synapse building [17–20, 37, 38]. Overexpression of syndecan-2 from DIV 1 to DIV 8 in hippocampal neurons disociated at E17-18 results in specific acceleration of morphological maturation of spines starting from preexisting filopodia: The fraction of mature-looking spines with heads increases from 20–25% in control to 75%, a value normally seen in DIV 30 hipoocampal cultures; moreover, the protrusions become shorter [17]. The density of protrusions remains at 20–25 per 100 μm dendrite [17]. Further, only a fraction of these syndecan-2 positive morphologically mature spines receives presynaptic boutons, highlighting the specific involvement of syndecan-2 in morphological maturation of dendritic protrusions, i.e. the postsynaptic terminal, but not in functional maturation of the synapse proper [17].

We found that DIV 5–10 and DIV 15–20 exposure of OTCs to Y-P30 results in a specific alteration of the density and morphology of dendritic protrusions along apical but not basal dendrites of pyramidal neurons (Fig 1C–1G). Between DIV 10 and DIV 20 dendritic protrusions on secondary oblique branches emerging from the apical dendrite nearly doubled in density, but remained lower in Y-P30-treated cultures compared to vehicle-treated controls with the effect being more pronouced at DIV 10 (Fig 1E). In contrast to dissociated hippocampal neurons [17], in cortical pyramidal cells in OTC, as early as DIV 10, the majority of protrusions were spines with heads suggesting a substantial difference in the time course of maturation as compared to dissociated cultures. The proportion of mushroom/stubby spines to thin spines remained unchanged. In vehicle-treated neurons we found 30.12 ± 3.61% mushroom/stubby spines and 69.48 ± 3.61% thin spines, and in Y-P30-treated neurons we found 29.99 ± 1.67% mushroom/stubby and 70.01 ± 1.67% thin spines (mean ± SEM at DIV 10 apical dendrites, total number of spines with heads set to 100%; n = 10 neurons for each condition). These values matched reported densities in vivo [32, 33]. Together this suggested that Y-P30 decelerates the building of new protrusions in apical dendrites. Apical dendrites are particularly sensitive because of higher amounts of calcium-permeable AMPA-receptors, which are involved in dendritic growth and spinogenesis [28]; moreover, they respond also to kainate-evoked network activity [30]. Importantly, major signaling mediators of syndecan-2, CASK and the syndecan-2 binding protein synbindin are enriched in apical dendrites and spines of neocortical pyramidal neurons (19, 37, 38]. Basal dendrites seemingly lack these players; and indeed basal dendrites employ growth mechanism which differ from those seen in apical dendrites [39]. Accordingly, we found that the protrusion density was unchanged in basal dendrites at DIV 10 and DIV 20 (Fig 1D). In line with the reported lack of syndecan-2 expression in interneurons [17, 37, 38], Y-P30 had no effect on the density of dendritic protrusions of interneurons analyzed in the same OTC (Fig 1D). At DIV 20, the proportion of protrusions with heads–mushroom/stubby and thin spines—was slightly higher, whereas the proportion of filopodia was significantly decreased in Y-P30-treated pyramidal cells compared to control (Fig 1F). Comparison of the longest decile of protrusions of secondary oblique branches revealed significantly shorter protrusions in Y-P30-treated neurons (Fig 1C and 1G). The shortening evoked by Y-P30 is in line with earlier reports on effects of enhanced syndecan-2 signaling on spine morphology [17, 37, 38].

### Y-P30 exposure does not alter expression of synaptic proteins

In view of the spinogenic but not synaptogenic effects of syndecan-2 enhancement, the observed Y-P30 triggered shift towards more mature spines warranted us to analyze the effect of this peptide on the expression of known synaptic proteins downstream of syndecans. The best characterized of such is CASK, which localizes to the synaptic membrane but also enters the nucleus to act as a cofactor for the transcription factor Tbr-1. In dissociated hippocampal neurons, Tbr-1 activates the expression of T-element containing genes relevant for synaptogenesis, including reelin, GluN1, GluN2B, and CK2α [40–42]. The actions of Y-P30 on CASK have been reported to be age-dependent; in DIV 8 dissociated cortical cells Y-P30 reduces nuclear CASK levels whereas at DIV 18, when syndecan-2 is more prominent, it increases nuclear CASK levels [16].

**Fig 4:**
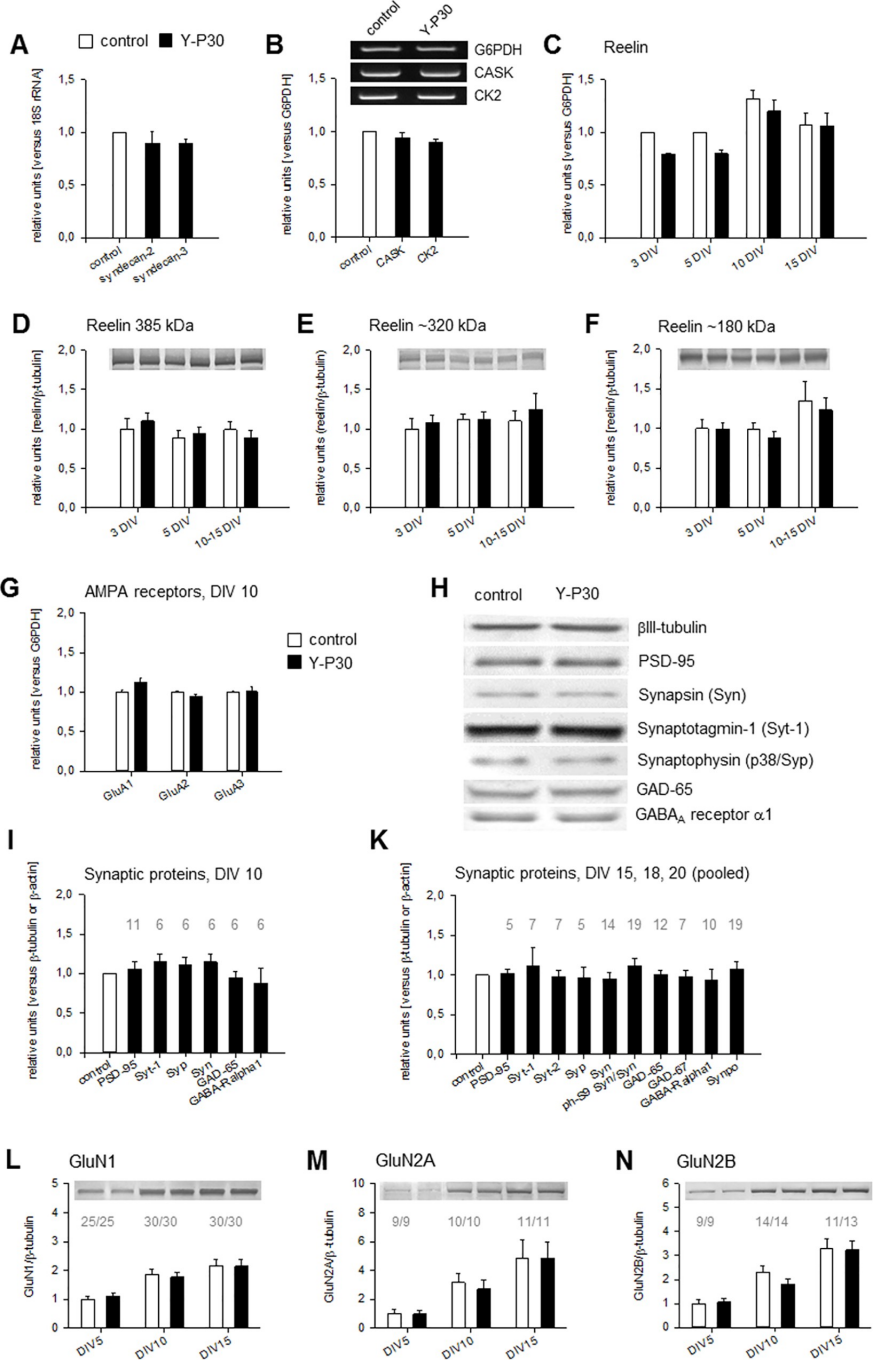
Y-P30 effects on expression of synaptic proteins. (A) Y-P30 did not alter the mRNA expression of syndecan-2/-3 and of (B) CASK and casein kinase 2α (CK2) as determined with qPCR. Mean ± SEM of four (A) and three (B) independent experiments normalized to the expression of glucose-6-phosphate dehydrogenase (G6PDH) or 18S rRNA. (C) Y-P30 initially decreased the mRNA expression of reelin at DIV 3–5, as determined with RT-PCR, but no longer at DIV 10 and DIV 15. Mean ± SEM of six to ten qPCR experiments with cultures from four independent preparations normalized to the expression of G6PDH. For DIV 3, control level at DIV 3 was set to 1. Expression levels at DIV 5, 10 and 15 were expressed relative to the DIV 5 control level which was set to 1. (D-F) The amount of the full length reelin protein at ~385 kDa (D) and the proteolytic fragments at 320 kDa (E) and 180 kDa (F) were not altered after stimulation of cortical cultures with Y-P30. Mean ± SEM of up to ten lysates from four independent preparations normalized to the expression of βIII-tubulin; average control level at DIV 3 was set to 1. (G) The mRNA expression of GluA1, GluA2, and GluA3 determined with RT-PCR was unchanged in Y-P30-treated cortical OTC. Mean ± SEM of more than 12 reactions run with 4–5 cDNA libraries each synthesized with the mRNA isolated from 5 OTC; the average control levels were set to 1. (H-K) The expression of the pre- and postsynaptic proteins synapsin-1, S9 phosphorylated synapsin, synaptotagmin-1, synaptotagmin-2, synaptophysin, synaptopodin, PSD-95, glutamate decarboxylase GAD65/67, and GABAA receptor α1 subunit was unchanged in OTC treated with Y-P30 until DIV 10 (H, I) or DIV 15, DIV 18, DIV 20 (pooled). Graphs in I, K show mean ± SEM, the number of lysates (1 OTC/lysate) probed for every protein is given above the bars; note that an equal number of vehicle-stimulated control OTC was run. OTC from 3–5 independent culture preparations; normalization to the expression of βIII-tubulin or β-actin on the same filter membrane to correct for loading. Average control levels were set to 1, but only one control bar for all has been plotted and therefore, a SEM is not indicated in the white bars of I and K. (L-N) Chronic exposure of OTCs to Y-P30 did not alter the developmental expression levels of GluN1, GluN2A and GluN2B protein, as seen at DIV 5, DIV 10 and DIV 15. Mean ± SEM, number of lysates probed for every protein is given above the bars; OTC from five to six independent preparations; normalization to βIII-tubulin; average of the vehicle-stimulated control levels at DIV5 were set to 1.

First, OTC were tested for syndecan expression, and both, syndecan-2 and syndecan-3 were expressed in DIV 10 cortical OTC (Fig 4A). This was similar to dissociated hippocampal cultures which also display syndecan-2 at DIV 9 [17]. In cortical OTC chronically exposed to Y-P30 the mRNA expression of syndecan-2 and syndecan-3 (Fig 4A) and of CASK and CK2α mRNA (Fig 4B) were unchanged. Reelin mRNA expression was decreased at DIV 3 and DIV 5 (Fig 4C), but no longer at DIV 10 and DIV 15 (expression relative to DIV 5 vehicle-stimulated control). However, protein blots for ~385 kDa full-length reelin and the ~320 kDa and ~180 KDa proteolytic fragments did not confirm a reduction because reelin remained at levels similar to vehicle-stimulated controls at DIV 3, DIV 5, and DIV 10–15 (Fig 4D–4F). The mRNA expression of the three major cortical AMPA receptor subunits GluA1, GluA2, and GluA3 was unchanged in Y-P30-treated cortical OTC at DIV 10 (Fig 4G). Y-P30 did not alter the expression of the pre- and postsynaptic proteins PSD-95, synapsin-1, synaptotagmin-1, synaptophysin, glutamate decarboxylases GAD-65/67 and the GABA_A_ receptor α1 and synaptopodin at DIV 10 (Fig 4H and 4I) and DIV 15–20 (Fig 4K). Synaptopodin is a marker for large-headed mature spines, and that synaptopodin levels were unchanged matched the observation that the ratio of mushroom/stubby-to-thin spines remained constant with Y-P30 treatment. Levels of growth-associated protein GAP-43, the astrocytic gial fibrillary acidic protein and the progenitor marker vimentin were also unchanged (in OTC exposed DIV 15–20). In OTC, levels of NMDA receptor subunits GluN1, GluN2A and GluN2B protein revealed the expected ontogenetic increase from DIV 5–15, however, protein expression levels in Y-P30-treated OTC were similar to vehicle-stimulated control OTC (Fig 4L, 4M and 4N).

### Y-P30 affects c-Src and GluN2B receptor phosphorylation

**Fig 5:**
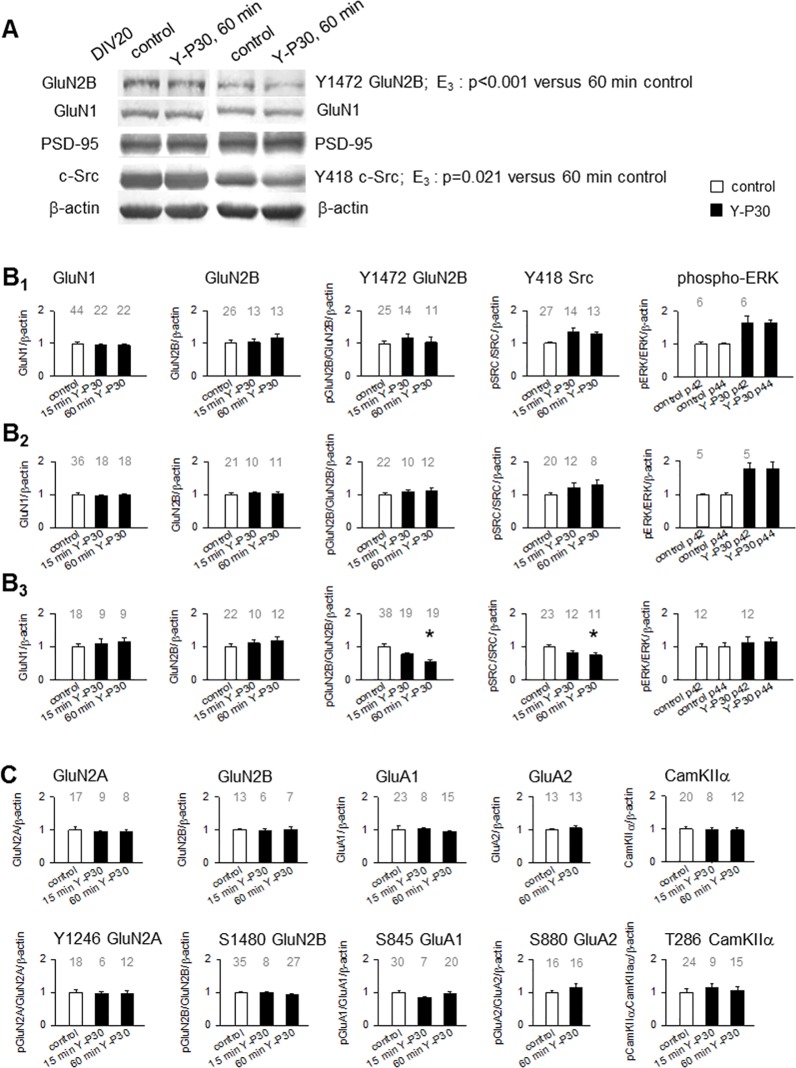
Effects of Y-P30 on expression and phosphorylation of glutamate receptors, Src, and ERK. (A-C) Qualitative (A) and quantitative (B, C) results of 15 and 60 min single-pulse treatment with Y-P30 at DIV 5, DIV 10 and DIV 20. (A) Representative blot at DIV 20; per lane, 50% of a lysate from a vehicle-treated control and a 60 min Y-P30-treated OTC was loaded on the left and the right side of a gel. Strips overlapping the target protein size range were cut after blotting from the nitrocellulose membrane and developed with antibodies to the indicated antigens. Note the reduction of Y1472 GluN2B and Y418 c-Src phosphorylation in the Y-P30-treated OTC (rightmost lane). (B_1_, B_2_, B_3_) Expression of GluN1, GluN2B, Y1472 phosphorylated GluN2B, Y418 phosphorylated c-Src, and phosphorylated p42/p44 ERK, at 15 min and 60 min after a single pulse of Y-P30. B_1_ top row at DIV 5; B_2_ middle row at DIV 10; B_3_ lower row at DIV 20. Note that p42/p44 ERK and c-Src phosphorylation were increased at DIV 5 and DIV 10. By contrast, at DIV 20, p42/p44 ERK was no longer activated, and Y418 c-Src and Y1472 GluN2B phosphorylation were decreased at 15 and at 60 min; Mann-Whitney U test p values given in (A). Mean ± SEM, number of lysates probed for every protein or phosphorylation site is given above the bars; vehicle-stimulated controls at the two time points have been pooled; OTC from five to six independent preparations. (C) Expression at DIV 20 of GluN2A and Y1246 GluN2A, GluN2B and S1480 GluN2B, GluA1 and S845 GluA1, GluA2 and S880 GluA2, and CamKIIα and T286 CamKIIα were not altered by a single pulse of Y-P30. Mean ± SEM, number of lysates probed for every protein or phosphorylation site is given above the bars; vehicle-stimulated controls at the two time points have been pooled; OTC from five to six independent preparations.

In immature neurons, acute stimulation with Y-P30 activates c-Src and p42/44 ERK phosphorylation [13, 14]. We therefore analyzed OTC stimulated for 15 min and 60 min with a single-pulse of 1 μM Y-P30 at DIV 20 and observed a decrease of Y1472 GluN2B and Y418 c-Src phosphorylation (Fig 5A). The finding let us to look for possible changes in expression and phosphorylation of synaptic proteins previously found to be downstream of syndecan-2 signaling during development at DIV 5 (Fig 5B_1_), DIV 10 (Fig 5B_2_) and DIV 20 (Fig 5B_3_). GluN1 and GluN2B proteins were not altered at the three ages (Fig 5B, first and second column). Tyrosine phosphorylation of Y1472 of GluN2B was not changed at DIV 5 and DIV 10, but Y-P30 evoked a fast dephosphorylation at DIV 20 (Fig 5A, Fig 5B, third column). The Y1472 phosphorylation is important to anchor GluN2B receptors to the postsynaptic membrane [43]. Phosphorylation of c-Src at the Y418 activation site was increased by Y-P30 at DIV 5 and 10 as previously reported [13], but at DIV 20, the Y–P30 treatment evoked a dephosphorylation (Fig 5A, Fig 5B, fourth column). Finally, phosphorylation of p42/p44 ERK was quickly activated at DIV 5 and DIV 10 as previously reported [13], but no longer at DIV 20 (Fig 5B, fifth column). Earlier studies showed that p42/p44 ERK activation by Y-P30 depends on c-Src [13] and thus, the run-down of c-Src activity could explain the absence of p42/p44 ERK activation at DIV 20.

At DIV 20, acute stimulation with Y-P30 did not change the expression and phosphorylation of GluN2A at Y1246 (Fig 5C, first column), GluN2B at S1480 (Fig 5C, second column), GluA1 at S880 (Fig 5C, third column), GluA2 (Fig 5C, fourth column). Finally, CaMKIIα phosphorylation at T286 (Fig 5C, fifth column) was not altered, and CaMKIIα has been implicated in the upregulation of Tbr-1 [42]. Together, this suggested a developmental switch in action targeting GluN2B phosphorylation in differentiated cortical neurons.

### Y-P30 depresses neurotrophin-3 expression

**Fig 6:**
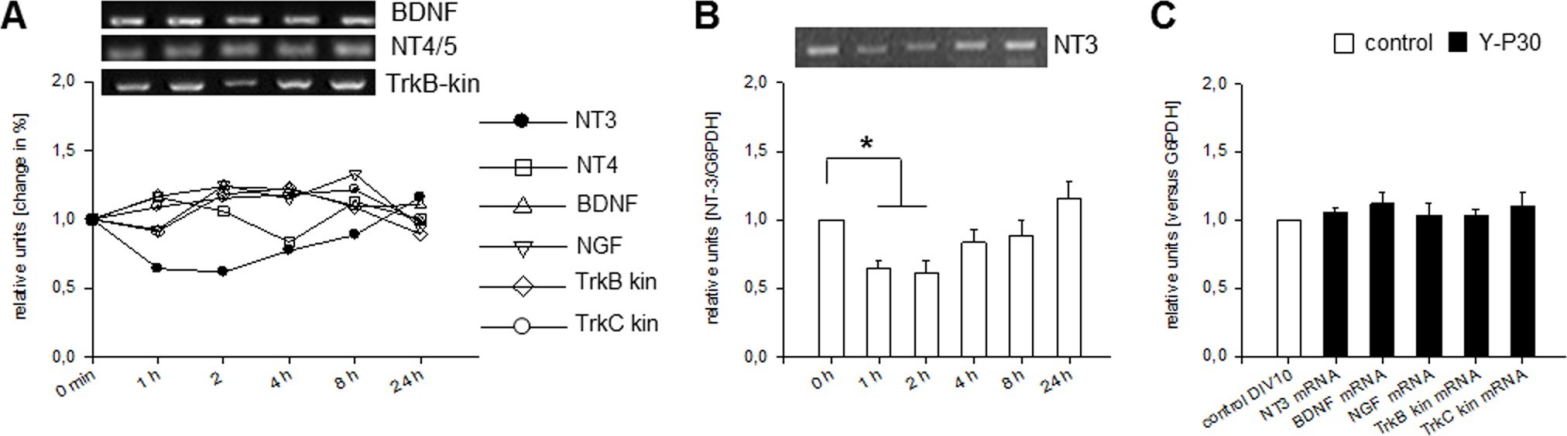
Y-P30 effects on neurotrophin expression. (A) Y-P30 did not alter the expression of BDNF, NT4, NGF, TrkB and TrkC (determined for the kinase domain-bearing full length receptors), but quickly reduced NT3 mRNA. (B) The expression of NT3 mRNA was reduced at 1 and 2 h after a single pulse of Y-P30. (C) Longterm treatment with Y-P30, 3 pulses from DIV 5 to DIV 10 followed by analysis at least 12 h after the last pulse did not alter the levels of trophic factor and receptor mRNAs. Shown are means (in A) and mean ± SEM (bar graphs in B, C) normalized to G6PDH of six RT-PCR reactions performed with three cDNA libraries per time point each synthesized with mRNA isolated from 5 OTC; untreated control (0 min) was set to 1, * p<0.05 at 1 and 2h, two-tailed t-test.

The spine phenotype led us to look at neurotrophin expression. Neurotrophins play major roles for spine formation and plasticity [25, 44]. Further, the alterations in Y-P30-triggered signaling might impact neurotrophin expression which is known to be regulated by activity including NMDA receptor signaling. This led us to study the mRNA expression of the four neurotrophins and the full-length receptors TrkB and TrkC in cDNA libraries synthesized with mRNA isolated from DIV 10 cortical OTC stimulated for 1, 2, 4, 8 and 24 h with a single pulse of Y-P30 (Fig 6). The levels of BDNF, NGF, NT4, TrkB and TrkC mRNA did not deviate significantly from the 0 min (untreated) time point (Fig 6A). A significant change was found for NT3 mRNA which was decreased for several hours after the Y-P30 pulse; it slowly recovered to control level within 24 hours (Fig 6A and 6B). Chronic treatment with Y-P30 followed by analysis minimum 12 h after the last pulse revealed NT3, BDNF, NGF and the full-length TrkB and TrkC receptor mRNA levels not different to levels in vehicle-stimulated control (Fig 6C). This suggested that pulse by pulse the synthesis of NT3 becomes transiently depressed.

### Lower frequency of glutamatergic mEPSC but no alteration of mEPSC amplitude and kinetics in cortical neurons exposed to Y-P30

**Fig 7:**
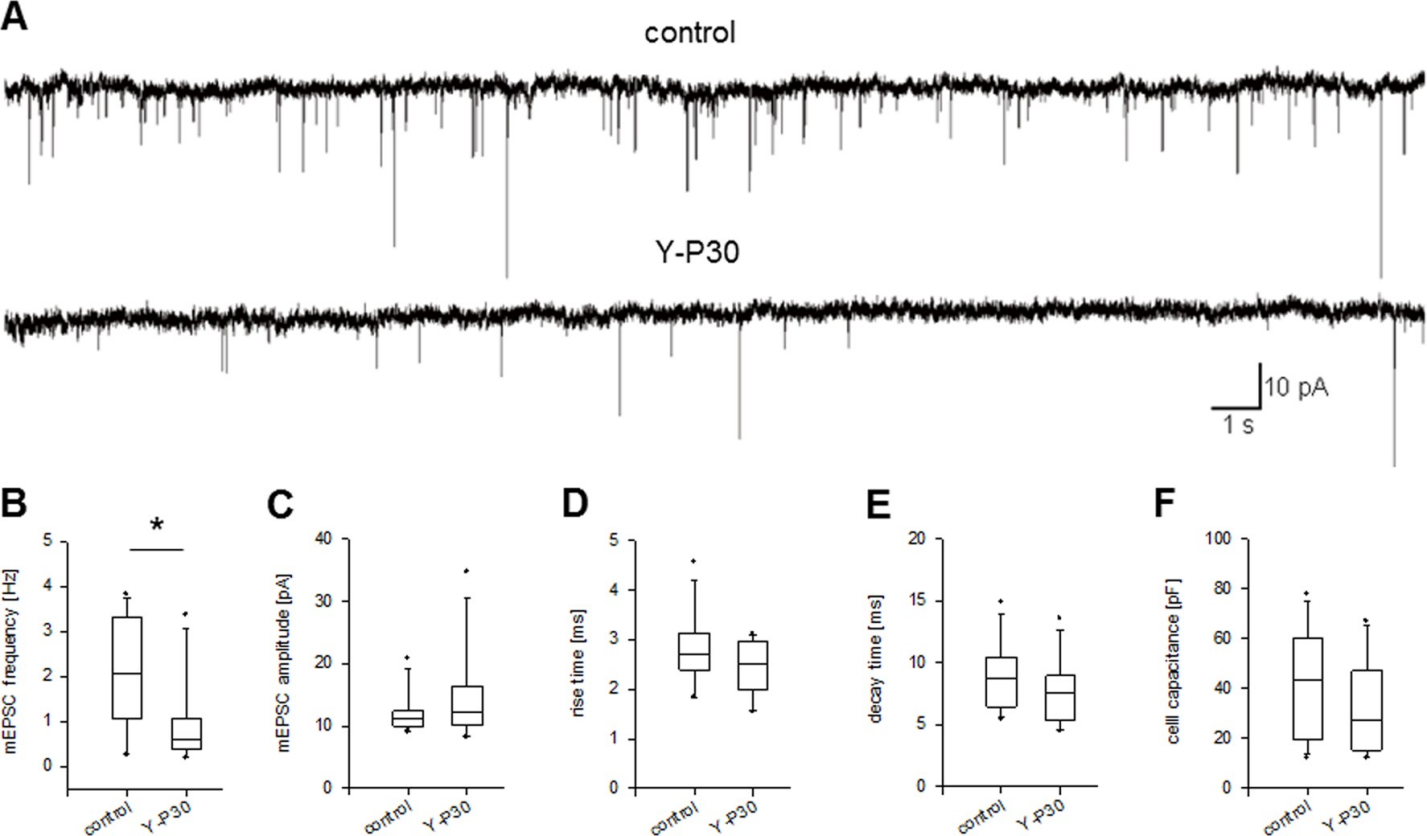
Lower frequency of glutamatergic mEPSCs but no alteration in mEPSC amplitude and kinetics in cortical pyramidal neurons exposed to Y-P30. (A) Sample traces of miniature excitatory postsynaptic currents (mEPSCs) recorded from DIV 15–17 dissociated cortical neurons under control conditions and following chronic exposure to Y-P30. (B) Recordings from neurons grown in the presence of Y-P30 revealed significantly lower mean mEPSC frequency, but not (C) mEPSC amplitude, (D) mEPSC rise time, or (E) mEPSC decay time. (F) Chronic exposure to Y-P30 did not have a significant effect on cell membrane capacitance. *p < 0.05, Mann-Whitney U-test.

NT3 is expressed and secreted by pyramidal neurons, it has a clear role in tuning up excitatory synapses by affecting presynaptic release [25], and exerts a unique function via TrkC receptors associated with heparan sulfated proteins at the excitatory presynapse [45–47]. Therefore, we figured that the presumably repetitive, albeit short-lasting, depression of NT3 transcription over a couple of days might have effects on the development of pre- or postsynaptic function. Patch clamp recordings from DIV 15–17 primary cortical neurons grown in presence of Y-P30 since DIV 7, demonstrated a two-fold lower frequency of non-NMDA receptor-mediated mEPSCs (Fig 7A and 7B). The postsynaptic parameters mEPSC amplitude rise and decay times were not changed (Fig 7C–7E). This, in view of the morphological data demonstrating that Y-P30 decreased the proportion filopodia-type protrusions generally thought not be synaptically functional, suggested an action on presynaptic function by Y-P30. In line with the observation that size and morphology of pyramidal neurons were not altered by Y-P30 treatment (see Fig 2, S1 Fig) no difference was seen in the membrane capacitance between Y-P30 and vehicle-treated neurons (Fig 7F).

### Lower protrusion densities are not reflected by changes of activity in OTC

The role of syndecan-2 mediated transition of dendritic filopodia to mature spines ist still not clear [18]. In dissociated neuron models the plating density of the cells influences the dendritogenesis and spinogenesis [17, 48]. For instance, in low density cultures excitatory synapses form on shafts rather than spines [48]. It has been suggested that the contact with axons plays a role in filopodia-to-spine transition, however, in syndecan-2 overexpressing hippocampal neurons the newly forming spines have not been contacted by presynaptic boutons [17]. Therefore, humoral factors like neurotrophins, or the presence of non-neuronal cells, or electrical activity can be equally involved. Calcium acting via CASK and mLIN towards NMDA receptors has been shown to play an important role in the filopodia-to-spine transition [18]. Arguing from another perspective, however, it is still debated if and how the making and breaking of spines impacts the cortical circuits [33].

**Fig 8:**
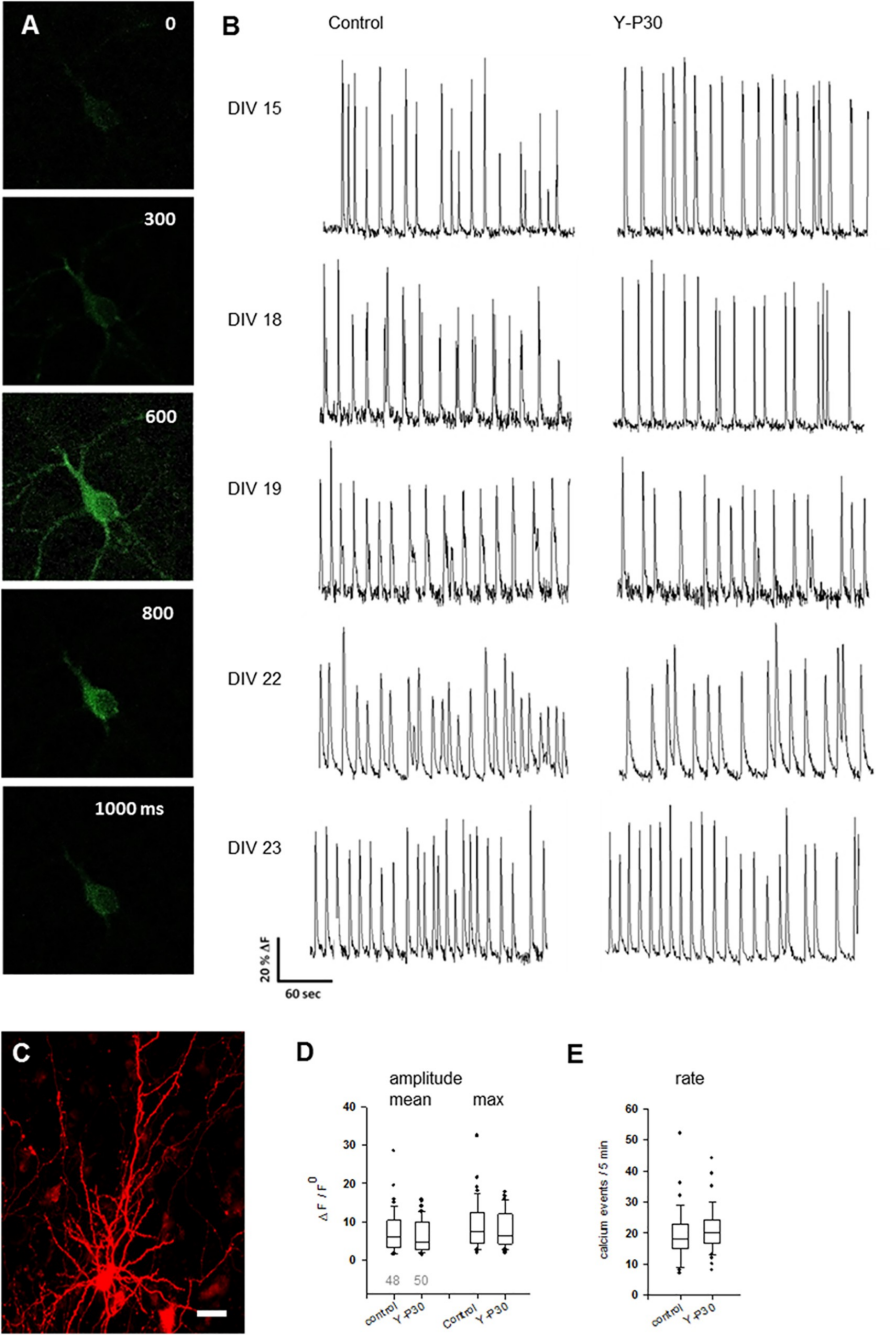
Rate and amplitude of calcium events are not affected in pyramidal neurons in OTC exposed to Y-P30. (A) GCaMP6m calcium signal of a DIV 14 pyramidal neuron. (B) Calcium event traces recorded at DIV 14–23 pyramidal neurons, vehicle-treated control and Y-P30 treated from DIV 8 with one pulse daily at 1 μM final concentration. (C) A representative cortical pyramidal cell at DIV 18 as identified by mCherry fluorescence, scale 20 μm. (D) Mean and maximal amplitude of calcium events were not different; Mann-Whitney U-test, p = 0.378 and p = 0.466 for mean and max amplitudes. (E) Rates of calcium events during a 5 min recording were not different, Mann-Whitney U-test, p = 0.126. Numbers in D indicate the number of total neurons recorded and plotted. Neurons were from 15 vehicle-treated and 14 Y-P30-treated OTC from 3 independent preparations.

This led us to record spontaneous calcium events, a proxy for neuronal action potential firing, in GCaMP6m-expressing neurons (Fig 8A and 8B) identified as pyramidal cells by co-transfected mCherry (Fig 8C) arbitrarily selected (in equal proportions) from supra- and infragranular layers in DIV 14–23 OTC grown in the presence of 1 μM Y-P30 from DIV 8. The time window matched the period where Y-P30 slows down the building of filopodia and of spines. However, neither mean nor maximal amplitudes (Fig 8D) nor the rate of calcium events during the 5 min recordings differed from those of neurons in vehicle-treated control OTC (Fig 8E). Together, the age-dependent difference in downstream signaling, the altered NT3 expression and GluN2B phosphorylation, differences in spine density, and reduced frequencies of mEPSC, apparently not impact the overall morphology of the neurons, the expression of pre- and postsynaptic proteins, and the network activity.

## Discussion

The main findings of our study are that Y-P30, a primate-specific peptide originating from the antimicrobial DCD gene expressed in eccrine sweat glands [4, 5] and also found in human breast milk [49], affects spine acquisition in cortical neurons. Exposure to Y-P30 slows down spine building resulting in a decreased density selectively of apical dendritic protrusions, decreases the proportion of filopodia, and promotes the shortening of the longest decile of protrusions. The decrease in protrusion density is unlikely to be explained by dendritic elongation because tree dimension of pyramidal cells and cell capacitance values were unaffected by Y-P30 treatment. Whole-cell recordings from principal neurons in cortical primary cultures grown in the presence of Y-P30 demonstrated no change in the amplitude of mEPSCs. Furthermore, the total protein levels of pre-and postsynaptic markers in cortical organotypic cultures were unaltered by Y-P30. This is in accord with the proposed mode of action of Y-P30 via enhancement of syndecan-2 signaling, which has been reported to specifically promote morphological but not functional maturation of spines [17].

The transient reduction of NT3 expression presently observed by Y-P30 was an unexpected and important finding, suggesting that syndecan signaling has an impact on neurotrophin expression. It is in line with the observed effects on spine density and mEPSC frequency in several aspects. Spine changes were seen at DIV 10 after just 3 days in the presence of Y-P30. At the same age, a single pulse of Y-P30 reduced NT3 mRNA expression for several hours without changing the expression of BDNF, NGF or NT4, or the full-length TrkB and TrkC receptors. NT3 expression is higher in fetal cortex and promotes the growth of cortical afferents. After birth, NT3 declines to levels much lower than for instance BDNF levels. The level of NT3 mRNA was not lastingly reduced after Y-P30 treatment, rather, we suggest that Y-P30 evokes, pulse by pulse, a transient reduction of NT3 transcription which normalizes within 12–24 h and did not lead to a longlasting deficit. However, it could lead to a transient impairment of NT3 signaling for several hours. NT3 is secreted in an activity-dependent manner, promotes LTP, and is a strong enhancer of excitatory connections [25, 50]. NT3 has no effect on interneurons because interneurons rarely express the cognate TrkC receptor [51]. First, a developmental delay scenario could be suggested because the frequency of mEPSC in pyramidal cells of rodent visual cortex increases steeply between P12 and P23 in vivo [52], and Y-P30 by decelerating spine acquisition could also slow down the development of functional presynapses. An intriguing function has recently been reported for TrkC receptors: They are selectively synaptogenic at glutamatergic synapses, a function not shared by TrkB and TrkA. Knockdown of TrkC in hippocampal neurons reduced excitatory synapse density, the frequency of mEPSC and the density of dendritic spines [45, 47, 53]. The receptor strengthens synapse adhesion by interacting via its extracellular domains with the presynaptically localized receptor tyrosine phosphatase sigma and with heparan-sulfated proteins [54, 55], and NT3 enhances these interactions in a concentration-dependent fashion, and, interestingly, the positive effect on presynaptic differentiation occurs within hours of exposure [45, 46, 53, 54, 55]. This suggested the following scenario. The Y-P30-induced decrease of NT3 could weaken TrkC receptor dimerization, reduce its interaction with the downstream interaction partners, and this way impair NT3/TrkC-mediated presynaptic differentiation, for instance, by decreasing vesicle recycling. An impairment of presynaptic differentiation and less efficient vesicle recycling could contribute to the observed decrease of mEPSC frequency in Y-P30-treated neurons. Indeed, NT3 and BDNF potentiate excitatory synapses by affecting the presynaptic release [25, 40]. Thus, transient reductions of NT3 might lead to presynaptic deficits. Although the levels of the presynaptic proteins presently analyzed were not altered, a subtle impairment of formation of presynaptic sites cannot be excluded. Moreover, we showed that the effect on protrusions occurs in just the apical dendritic domain; protrusions on basal dendrites and interneurons were unaffected.

Y-P30 did not alter total protein levels of pertinent postsynaptic markers e.g. glutamate receptor proteins, in line with mEPSC amplitudes being not altered. Also, for instance, the expression of synaptopodin was unchanged, and indeed, Y-P30 did not affect the ratio of mushroom/stubby-to-thin spines. Intriguingly however, at DIV 20 in the most mature neurons analyzed until now, we observed a selective depression of Src family kinase phosphorylation and Y1472 GluN2B phosphorylation. Y1472 phosphorylation does not affect NMDA receptor-mediated currents, but has been shown to be critical for anchoring GluN2B-containing receptors at the postsynaptic density zone [43]. In hippocampal neurons, Y1472 GluN2B hyperphosphorylation correlates with higher density and higher motility of dendritic filopodia [56], with longterm potentiation [57, 58], and brain injury [59]. In contrast, Y1472 hypophosphorylation has been shown to liberate surface-expressed GluN2B-containing receptors from the anchor proteins, such that GluN2B-containing receptors can diffuse to the synaptic periphery, which increases the probability for their endocytosis. This could subtly contribute to a switch from GluN2B-containing NMDA receptors, which are associated with an increased ability for plastic changes, to GluN2A-containing NMDARs, which stabilize spines. Indeed, a decrease in Y1472 GluN2B phosphorylation correlates with an enhanced activation of synaptic GluN2A receptors [60].

Interestingly, despite these changes and a quite substantial deficit in the density of mushroom/stubby and thin spines we could not detect any difference in spontaneous action potential firing in pyramidal neurons in OTC tested from DIV 14 to DIV 23. It might be due to the spatially selective protrusion deficit. In fact, the basal dendrites dominate the dendritic tree and receive a substantial portion of all synaptic input and a majority of the intracortical synapses from horizontal and columnar circuits. Basal dendritic morphology and spine equipment was not altered after Y-P30 treatment, presumably because basal dendrites are not enriched with the syndecan-2 signaling machinery. By contrast, the apical dendrites are equipped with the signaling maschinery downstream of syndecan-2, and apical dendrites of DIV 10 pyramidal neurons are particularly sensitive to an increase in network activity [30] which together might represent the basis for an enhanced spine plasticity. However, it has been argued that the appearance and disappearance of dendritic protrusions might have little or no impact on the brain circuit [33].

As an outlook, the fact that dermcidin is secreted by skin and present in breast milk leads to the question of whether Y-P30 from the environment can enter the brain of the newborn. Here, an interesting observation is that after injection of tagged Y-P30 peptide into the rat maternal circulation, the peptide could be detected using anti-tag antibodies in brain of the offspring [12]. In this view, our results could suggest the presence of a molecular mechanism bridging infant neural plasticity and primate social interaction, particularly between mother and suckling.

## Conclusion

The peptide Y-P30 derives from the primate-specific dermcidin precursor protein expressed mainly by eccrine sweat glands. Previously we reported that Y-P30 is an activating ligand of neuronal syndecans-2/3. Syndecan-3 is enriched in axons, and Y-P30 promotes axon elongation. Syndecan-2 preferentially localizes at dendritic spines. In the present work, chronic exposure of rat cortical pyramidal neurons to Y-P30, akin to activation of syndecan-2 signaling, decreases filopodial density and slows down acquisition of dendritic protrusions but promotes the morphological maturation of dendritic spines. Notably, Y-P30 had no effect on spines of cortical non-pyramidal neurons–a neuronal population known to lack syndecan-2.

## Supporting information

S1 Table Primary antibodies and reagents. List of antibodies used in the study, with the order numbers of the companies. Sources are: Calbiochem via Merck Chemicals GmbH, Darmstadt, Germany; Cell Signaling, Frankfurt, Germany; Chemicon via Merck Chemicals GmbH, Darmstadt, Germany; Clontech via Takara Bio Europe, Saint-Germain-en-Laye, France; Dako, Hamburg, Germany; DSHB, Developmental Studies Hybridoma Bank, Iowa City, USA; Enzo Life Science, Lörrach, Germany; Invitrogen/Thermo Fisher Scientific/Life Technologies GmbH, Darmstadt, Germany; Merck Millipore, Darmstadt, Germany; NeuroMab, UC Davis, CA, USA; Peprotech, Hamburg, Germany; Promega, Mannheim, Germany; Rockland Inc. via Biomol, Hamburg, Germany; Sigma, Deisenhofen, Germany; Santa Cruz Biotechnology, Heidelberg, Germany; SySy, Synaptic Systems, Göttingen, Germany. Abbreviations: AP, alkaline phosphatase; dk, donkey; gp, guinea pig; gt, goat; HRP, horseradish peroxidase; ms, mouse; rb, rabbit.
(DOCX)

S2 Table PCR primers, amplicons and reaction conditions. (DOCX)

S1 Fig Morphometry of pyramidal cells. (A-D) Pyramidal cells from layers II/III and (E-H) pyramidal cells from layers V/VI (excluding layer V corticotectal cells with tufts in layer I) were reconstructed at DIV 5, DIV 10 and DIV 20. OTC were exposed to 1 μM Y-P30 applied with fresh medium at DIV 2 and 4 for analysis at DIV 5, at DIV 7 and 9 for analysis at DIV 10, or daily from DIV 15 for analysis at DIV 20. Control OTC were vehicle -treated with 5 mM Tris-HCl pH 7.4. Values, SEM and the number of cells are given in Fig 2. At DIV 5, only cells from infragranular layers were analyzed because supragranular neurons are too immature to reliably identify the type.
(TIF)

S2 Fig Morphometry of multipolar interneurons. Cells reconstructed from DIV 10 and DIV 20 OTC exposed to 1 μM Y-P30 applied with fresh medium at DIV 7 and 9 for analysis at DIV 10, or daily from DIV 15 for analysis at DIV 20 (same OTC delivering the pyramidal cell data). Control OTC were vehicle treated with 5 mM Tris-HCl pH 7.4. Mean ± SEM for (A) total dendritic length; (B) total dendritic segments; (C) mean dendritic length; (D) mean dendritic segment number; (E) number of primary dendrites/neuron. Values, SEM and the number of cells are given in Fig 3. (F) Soma area of GABA-ir neurons was also not influenced by Y-P30. The 20 ng/mL medium NT4 treatment has been done as positive control (12 OTC) to show responsiveness of interneurons. ANOVA on ranks followed by Mann-Whitney U-test versus DIV 10 control: p<0.05.
(TIF)
