# Supplementary material for: The primate-specific peptide Y-P30 regulates morphological maturation of neocortical dendritic spines
Source: PLoS One. 2019 Feb 13;14(2):e0211151. doi: 10.1371/journal.pone.0211151 (PMC6373909; doi:10.1371/journal.pone.0211151)
Supplement: S1 Table — List of antibodies used in the study, with the order numbers of the companies. Sources are: Calbiochem via Merck Chemicals GmbH, Darmstadt, Germany; Cell Signaling, Frankfurt, Germany; Chemicon via Merck Chemicals GmbH, Darmstadt, Germany; Clontech via Takara Bio Europe, Saint-Germain-en-Laye, France; Dako, Hamburg, Germany; DSHB, Developmental Studies Hybridoma Bank, Iowa City, USA; Enzo Life Science, Lörrach, Germany; Invitrogen/Thermo Fisher Scientific/Life Technologies GmbH, Darmstadt, Germany; Merck Millipore, Darmstadt, Germany; NeuroMab, UC Davis, CA, USA; Peprotech, Hamburg, Germany; Promega, Mannheim, Germany; Rockland Inc. via Biomol, Hamburg, Germany; Sigma, Deisenhofen, Germany; Santa Cruz Biotechnology, Heidelberg, Germany; SySy, Synaptic Systems, Göttingen, Germany. Abbreviations: AP, alkaline phosphatase; dk, donkey; gp, guinea pig; gt, goat; HRP, horseradish peroxidase; ms, mouse; rb, rabbit. (DOCX) [file pone.0211151.s001.docx]

**S1 Table. Primary antibodies and reagents.**

| **Primary antibody**  **against** | **Species; Source** | **Primary antibody**  **against** | **Species; Source** |
| --- | --- | --- | --- |
| GAD-65/67 | ms; 1:5000; Enzo Life Sci,  #ADI-MSA-225-E | ph S9 Synapsin | rb; 1:1000; Rockland,  #612-401-C93 |
| Synapsin-1 | ms; 1:3000; SySy, #106 001 | ph Y1246 GluN2A | rb; 1:1000; Cell Signaling,  #4206 |
| GABA_A_Rα1 | gp; 1:1000; SySy, #224 204 | ph S845 GluA1 | rb; 1:1000; Rockland Inc.  #612-401-C83 |
| Synaptopodin | rb; 1:500; SySy, #163 002 | ph S880 GluA2 | rb; 1:1000; Rockland Inc.  #612-401-D64 |
| PSD-95 | rb; 1:1000; SySy, #124 002 | ph S1480 GluN2B | rb; 1:1000; Rockland Inc.  #612-401-D93 |
| Synaptotagmin-1 | mo; 1:1000; SySy, #105 001 | ph Y1472 GluN2B | rb; 1:1000; Calbiochem,  #454583 |
| Synaptophysin-1 | ms; 1:1000; SySy, #101 011 | GluA1 | ms; 1:1000; NeuroMab  #75-327 |
| p42/44 ERK/MAPK | rb; 1:1000; Santa Cruz, #K-23 | GluA2 | ms; 1:1000; NeuroMab  #75-002 |
| Src kinase | rb; 1:1000; Santa Cruz, #sc-18 | ph Y418 Src | rb; 1:1000; Invitrogen, #44660G |
| Vimentin | ms; 1:3000; Sigma, #V6630 | GABA | ms; 1:2000; Chemicon, #MAB316 |
| double-phosph. ERK/ MAPK T183, Y185 | ms; 1:1000; Sigma, #M8159 | Reelin | ms; 1: 1000; Chemicon, #G10 |
| EGFP | ms; 1:1500; Sigma, #G6539 | Reelin | ms; 1: 1000; DSHB, #E4 |
| β -tubulin | ms; 1:500; Sigma, #T5293 | GFAP | rb; 1:500; Dako, #Z0334 |
| β -actin | ms; 1:1000; Sigma, #A1978 | GluN1 | rb; 1:1000; Merck Millipore #AB9864 |
| CaMKIIα | ms; 1:2000; Sigma, #C265 | GluN2B | ms; 1:1000; Merck Millipore  #06-600 |
| ph T286 CaMKIIα | rb; 1:2000, Promega, #V1111 | GluN2A | rb; 1:1000; Merck Millipore  #07-632 |
| **Other reagents** | **Source** | **Other reagents** | **Source** |
| anti mouse, AP | rabbit; 1:1500; Dako | anti goat, AP | dk; 1:1000; Promega |
| anti guinea pig | rabbit; 1:1500; Dako | anti mouse, biotin | gt; 1:1000; DAKO |
| anti rabbit, biotin | goat; 1:1000; Dako | avidin biotin HRP | 1:1500; Dako |
| anti rabbit, AP | goat; 1:1500; Dako | streptavidin AP | 1:1000; Dako |
| normal goat serum | gt; 5%; Dako | NT4 | 20 ng/mL, Peprotech |
| pEGFP-N1 in pcDNA3.0 | Clontech, cat# 632370 | pmCherry in pcDNA3.0 | Clontech, cat# 632523 |
| pGP-CMV-GCaMP6m | Addgene, cat# 40754 |  |  |

**S1 Table. Primary antibodies and reagents.** List of antibodies used in the study, with the order numbers of the companies. Sources are: Addgene, Watertown, MA, USA; Calbiochem via Merck Chemicals GmbH, Darmstadt, Germany; Cell Signaling, Frankfurt, Germany; Chemicon via Merck Chemicals GmbH, Darmstadt, Germany; Clontech, Heidelberg, Germany, via Takara Bio Europe, Saint-Germain-en-Laye, France; Dako, Hamburg, Germany; DSHB, Developmental Studies Hybridoma Bank, Iowa City, USA; Enzo Life Science, Lörrach, Germany; Invitrogen/Thermo Fisher Scientific/Life Technologies GmbH, Darmstadt, Germany; Merck Millipore, Darmstadt, Germany; NeuroMab, UC Davis, CA, USA; Peprotech, Hamburg, Germany; Promega, Mannheim, Germany; Rockland Inc. via Biomol, Hamburg, Germany; Sigma, Deisenhofen, Germany; Santa Cruz Biotechnology, Heidelberg, Germany; SySy, Synaptic Systems, Göttingen, Germany. Abbreviations: AP, alkaline phosphatase; dk, donkey; gp, guinea pig; gt, goat; HRP, horseradish peroxidase; ms, mouse; rb, rabbit.
