# Supplementary material for: The primate-specific peptide Y-P30 regulates morphological maturation of neocortical dendritic spines
Source: PLoS One. 2019 Feb 13;14(2):e0211151. doi: 10.1371/journal.pone.0211151 (PMC6373909; doi:10.1371/journal.pone.0211151)
Supplement: S2 Table — (DOCX) [file pone.0211151.s002.docx]

**Supporting Information**

**S2 Table. PCR primers, amplicons and reaction conditions.**

| Target | amplicon, conditions | Reference |
| --- | --- | --- |
| Syndecan-2 | for 5´-ACCCACTCAGACCGAGTCAC-3`  rev 5´-CTAGAACTTCCGTCCGCTTG-3´ , 149 bp | Tai et al., 2000 |
| Syndecan-3 | for 5´-CACCTCCTCCCAGGGAAAG-3´  rev 5´-ACGCTAGAGTGGCCTCCCTTA-3´ , 69 bp | Tai et al., 2000 |
| Reelin | for 5´-CAGCGTCAACAACGGCATCACG-3´  rev 5´-TGCCACCAGCGCAGCAAAAC-3´, 132 bp | n.a. |
| 18S RNA ribosomal | for 5´-CATGGTGACCACGGGTGAC-3’  rev 5´-TTCCTTGGATGTGGTAGCCG-3´, 78 bp | n.a. |
| G6PDH (qPCR) | for 5´-CTAGCTGCTGCTCTTACCTTCC-3´  rev 5´-TGCTACTTGACATTCCTCCTCA-3´, 160 bp | Ho et al., 1988 |
| BDNF | bp 158–393; 30 cycles: 1 min 94 °C, 1 min 55 °C, 1 min 72 °C | Maisonpierre et al., 1990 |
| NT3 | bp 158–634; 36 cycles: 1 min 94 °C, 1 min 55 °C, 2 min 72 °C | Maisonpierre et al., 1990 |
| NT4 | bp 309–605; 36 cycles: 1 min 94 °C, 1 min 57.5 °C, 1 min 72 °C | Berkemeier et al., 1991 |
| NGF | bp 456–946; 33 cycles: 1 min 94 °C, 1 min 57 °C, 2 min 72 °C | Ming et al., 1999 |
| TrkB kinase | bp 1422–1931; 30 cycles: 1 min 94 °C, 1 min 55 °C, 1 min 72 °C | Middlemas et al., 1991 |
| TrkC kinase | bp 1459-1943; 30 cycles: 1 min 94 °C, 1 min 55 °C, 1 min 72 °C | Valenzuela et al., 1993 |
| GluA1 | bp 2290-2554; 35 cycles: 1 min 94°C, 1 min 55°C, 1 min 72°C | n.a. |
| GluA2 | bp 2122-2353; 35 cycles: 1 min 94°C, 1 min 55°C, 1 min 72°C | n.a. |
| GluA3 | bp 2162-2634; 35 cycles: 1 min 94°C, 1 min 55°C, 1 min 72°C | n.a. |
| CASK | bp 2415-2967; 33 cycles: 20 s 94°C, 1 min 59°C, 1 min 68 °C | n.a. |
| CK2α | bp 749-1093; 35 cycles: 20 s 94°C, 1 min 59°C, 1 min 68°C | n.a |
| G6PDH (RT-PCR) | bp 2112–2272; 30 cycles: 1 min 94 °C, 1 min 55 °C, 1 min 72 °C | Ho et al., 1988 |

**S2 Table. PCR primers, amplicons and reaction conditions.** Used to amplify the target mRNAs analyzed; bp, base pair length of amplicon, n.a., amplicons selected and primers designed in the lab with appropriate software tools.

**References**

Berkemeier LR, Winslow JW, Kaplan DR, Nikolics K, Goeddel DV, Rosenthal A. Neurotrophin-5: a novel neurotrophic factor that activates trk and trkB. Neuron. 1991;7:857-66. pmid:1742028

Ho YS, Howard AJ, Crapo JD. Cloning and sequence of a cDNA encoding rat glucose-6-phosphate dehydrogenase. Nucleic Acids Res 1998;16:7746. pmid:3412913

Maisonpierre PC, Belluscio L, Friedman B, Alderson RF, Wiegand SJ, Furth ME et al. NT-3, BDNF, and NGF in the developing rat nervous system: parallel as well as reciprocal patterns of expression. Neuron. 1990; 5:501–9. pmid:1688327

Middlemas DS, Lindberg RA, Hunter T. TrkB, a neural receptor protein-tyrosine kinase: evidence for a full-length and two truncated receptors. Mol Cell Biol. 1991;11:143–53. pmid:1846020

Ming Y, Bergman E, Edström E, Ulfhake B () Reciprocal changes in the expression of neurotrophin mRNAs in target tissues and peripheral nerves of aged rats. Neurosci Lett. 1999;273:187–90. pmid:10515190

Tai JT, Brooks EE, Liang S, Somogyi R, Rosete JD, Lawn RM et al. Determination of temporal expression patterns for multiple genes in the rat carotid artery injury model. Arteriosclerosis, Thrombosis, Vasc Biol. 2000 20:2184–91. pmid:11031202

Valenzuela DM, Maisonpierre PC, Glass DJ, Rojas E, Nuñez L, Kong Y et al. Alternative forms of rat TrkC with different functional capabilities. Neuron 1993;10:963–74. pmid:8494647
