# Supplementary figures and images for: The primate-specific peptide Y-P30 regulates morphological maturation of neocortical dendritic spines
Source: PLoS One. 2019 Feb 13;14(2):e0211151. doi: 10.1371/journal.pone.0211151 (PMC6373909; doi:10.1371/journal.pone.0211151)

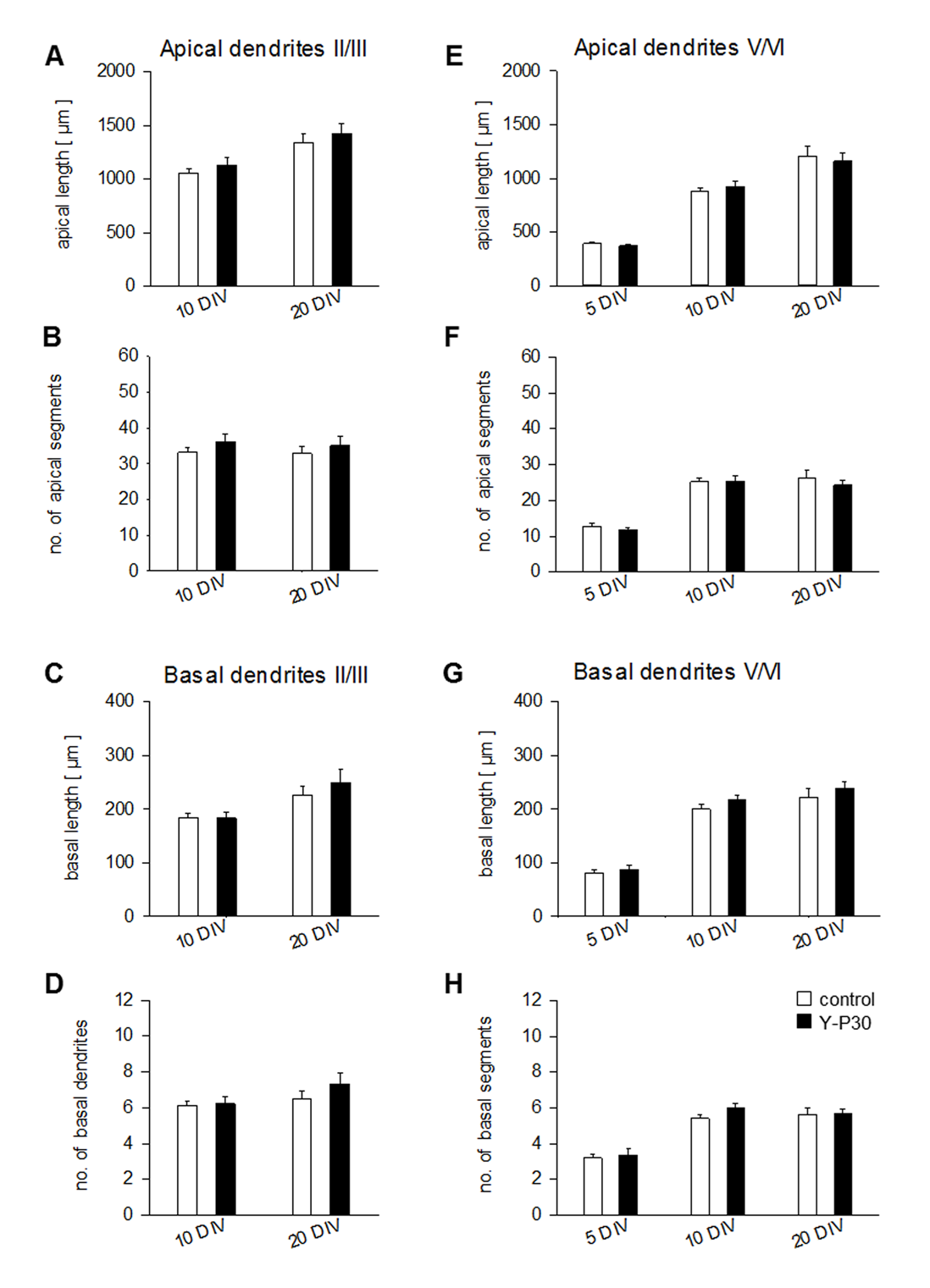

Supplement: S1 Fig — (A-D) Pyramidal cells from layers II/III and (E-H) pyramidal cells from layers V/VI (excluding layer V corticotectal cells with tufts in layer I) were reconstructed at DIV 5, DIV 10 and DIV 20. OTC were exposed to 1 μM Y-P30 applied with fresh medium at DIV 2 and 4 for analysis at DIV 5, at DIV 7 and 9 for analysis at DIV 10, or daily from DIV 15 for analysis at DIV 20. Control OTC were vehicle -treated with 5 mM Tris-HCl pH 7.4. Values, SEM and the number of cells are given in Fig 2. At DIV 5, only cells from infragranular layers were analyzed because supragranular neurons are too immature to reliably identify the type. (TIF) [file pone.0211151.s003.tif]

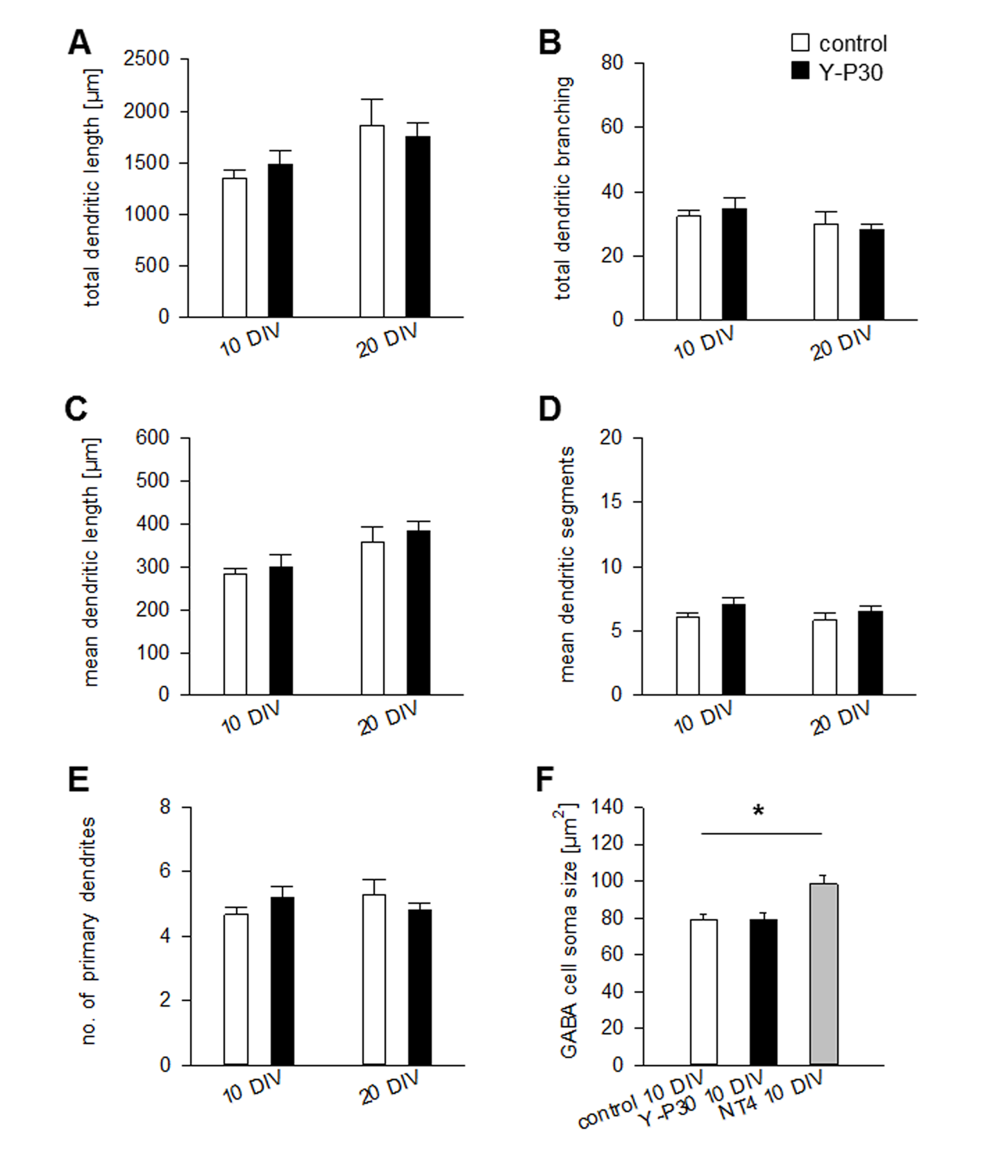

Supplement: S2 Fig — Cells reconstructed from DIV 10 and DIV 20 OTC exposed to 1 μM Y-P30 applied with fresh medium at DIV 7 and 9 for analysis at DIV 10, or daily from DIV 15 for analysis at DIV 20 (same OTC delivering the pyramidal cell data). Control OTC were vehicle treated with 5 mM Tris-HCl pH 7.4. Mean ± SEM for (A) total dendritic length; (B) total dendritic segments; (C) mean dendritic length; (D) mean dendritic segment number; (E) number of primary dendrites/neuron. Values, SEM and the number of cells are given in Fig 3. (F) Soma area of GABA-ir neurons was also not influenced by Y-P30. The 20 ng/mL medium NT4 treatment has been done as positive control (12 OTC) to show responsiveness of interneurons. ANOVA on ranks followed by Mann-Whitney U-test versus DIV 10 control: p<0.05. (TIF) [file pone.0211151.s004.tif]
